# Transcriptomics Integrated With Widely Targeted Metabolomics Reveals the Mechanism Underlying Grain Color Formation in Wheat at the Grain-Filling Stage

**DOI:** 10.3389/fpls.2021.757750

**Published:** 2021-10-14

**Authors:** Li Li, Zhiyou Kong, Xiuju Huan, Yeju Liu, Yongjiang Liu, Qianchao Wang, Junna Liu, Ping Zhang, Yirui Guo, Peng Qin

**Affiliations:** ^1^College of Agronomy and Biotechnology, Yunnan Agricultural University, Kunming, China; ^2^College of Natural Resources and Environment, Baoshan University, Baoshan, China; ^3^Graduate Office, Yunnan Agricultural University, Kunming, China

**Keywords:** color wheat, grain, color formation, grain-filling stage, metabolomic, transcriptomic

## Abstract

Colored wheat grains have a unique nutritional value. To elucidate the color formation mechanism in wheat seeds, comprehensive metabolomic and transcriptomic analyses were conducted on purple (Dianmai 20-1), blue (Dianmai 20-8), and white (Dianmai 16) wheat at the grain-filling stage. The results showed that the flavonoid biosynthesis pathway was closely related to grain color formation. Among the 603 metabolites identified in all varieties, there were 98 flavonoids. Forty-six flavonoids were detected in purple and blue wheat, and there were fewer flavonoids in white wheat than in colored wheat. Integrated transcriptomic and metabolomic analyses showed that gene expression modulated the flavonoid composition and content, resulting in different metabolite levels of pelargonidin, cyanidin, and delphinidin, thus affecting the color formation of wheat grains. The present study clarifies the mechanism by which pigmentation develops in wheat grains and provides an empirical reference for colored wheat breeding.

## Introduction

Wheat (*Triticum aestivum* L.) is a gramineous plant, accounts for about 20% of the total caloric intake of human beings (Chen et al., 2020), and is a major global food source. The grains of most wheat varieties cultivated worldwide are amber (white) in color. However, Wheat cultivars with different grain colors have attracted the interest of scientists and food processors because they are rich in anthocyanins and other nutrients (Saini et al., 2020). The wheat seed coat contains numerous anthocyanins, proanthocyanidins, and flavonols. Flavonol is a key precursor of seed coat pigment (Czemmel et al., 2009). Seed pigmentation affects grain appearance and quality and protects the seed against microbial pathogens, insect attack, and ultraviolet light (Wan et al., 2020). Anthocyanins provide a certain measure of protection against cancers, cardiovascular, and other diseases (Butelli et al., 2008). Colored wheat varieties generally have higher protein, essential amino acid, and total amino acid levels than common wheat (Tian et al., 2018). Prior studies have shown that the consumption of black wheat reduces fat levels and mitigates obesity co-morbidities more effectively than the consumption of common wheat (Sharma et al., 2020) and that blue wheat regulates blood sugar levels in patients with diabetes (Tyl and Bunzel, 2014).

**FIGURE 1:**
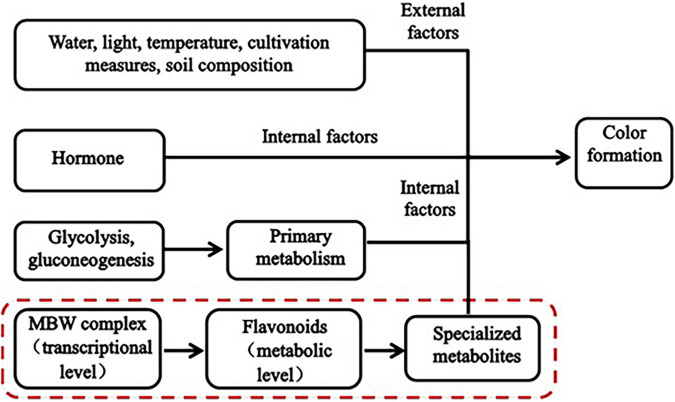
Mechanism underlying plant color formation.

Plant color formation has become a research hotspot, is affected by both internal and external factors (Figure 1), and is regulated by glycolysis and gluconeogenesis (primary metabolism), the flavonoid biosynthesis pathway (specialized metabolism), and certain transcription factors (TFs). Numerous studies have shown that flavonoids are the main cause of plant color formation. Flavonoids occur widely in the plant kingdom, and approximately 9,000 have been identified in plants to date (Ferrer et al., 2008). Different flavonoids have been detected in food crops, ornamentals, and medicinal plants (Yonekura-Sakakibara and Saito, 2014). In total, 188 flavonoids have been identified in four pepper varieties with different fruit colors. In purple pepper, the expression of flavonoids and regulatory genes is highly upregulated (Liu et al., 2020). Flavonoid biosynthesis is regulated at the transcriptional level mainly by the MBW (MYB-bHLH-WD40) complex (Xu et al., 2015). Myeloblastosis (MYB) TFs, basic helix-loop-helix (bHLH), and WD40 repeat proteins (WDR) form the MBW (MYB-bHLH-WD40) complex. The bHLH TFs form bridges with WDR and promote anthocyanin biosynthesis (Hichri et al., 2010). The expression of flavonoid metabolites and several MYB TFs is induced by injury and oxidation in rose. RRMYB5 and RRMYB10 regulate flavonoid biosynthesis in rose and play key roles in the feedback loop responding to injury and oxidation (Shen et al., 2019). Flavonoids comprise flavanones, flavonols, flavanols, anthocyanins, and isoflavones (Lepiniec et al., 2006). Anthocyanins are the main color constituents in plants. That is main flavonoids of anthocyanins is affecting the formation of plant color. Variations in anthocyanin composition and concentration are primarily responsible for color differences among plants (Baranac et al., 1997). Anthocyanins bind to sugars to form glycosides. Anthocyanin accumulation is regulated by various genes, such as chalcone synthase (CHS), chalcone isomerase (CHI), flavanone 3-hydroxylase, flavonoid 30-hydroxylase, and anthocyanin synthase. CHS is the enzyme catalyzing the first step in the flavonoid biosynthesis pathway; it catalyzes the synthesis of naringenin chalcone from 4-coumaroyl-CoA and malonyl-CoA. (Yan et al., 2020). Anthocyanin biosynthesis varies with cultivar, phytohormone level, and sugar accumulation. This variation results in color differences (Teng et al., 2005). The anthocyanin content in purple turnip is higher than that in green turnip, these cultivars differ in terms of the expression of certain structural genes in the flavonoid biosynthesis pathway, MYB, bHLH, and several key members of the WRKY family (Zhuang et al., 2019). Nineteen key MYB regulatory factors and anthocyanin biosynthesis genes are co-expressed during leaf color development in two different *Lagerstroemia* varieties (Qiao et al., 2019). Certain studies have demonstrated that plant pigment formation is associated with flavonoids and anthocyanins. Phytohormones also affect plant color formation to some extent. Ethylene inhibits anthocyanin biosynthesis, while jasmonic acid promotes anthocyanin and flavonoid/isoflavone biosynthesis in red pear fruit (Ni et al., 2020). Water, light, temperature, cultivation practices, soil composition and other external factors affect the biosynthesis of anthocyanins to indirectly influence the color development of plants (Yang et al., 2020). Dong et al. (2019) showed that several anthocyanin biosynthesis genes and at least five TFs significantly differed between green and purple asparagus varieties. Moreover, anthocyanin accumulation in asparagus was dependent on light. Anthocyanin accumulation is also influenced by temperature and light exposure. The pulp of blood orange fruit accumulates anthocyanins in response to cold induction. When light exposure is adequate, however, the pericarp accumulates anthocyanins (Huang et al., 2019). A previous transcriptomic analysis revealed that ultraviolet light significantly affects anthocyanin accumulation in wheat (Wang et al., 2019). Water also influences anthocyanin accumulation. In grape, controlled deficit irrigation increased anthocyanin monomer content (Yang et al., 2020).

Typically, biological processes are complex and interactive. No single dataset can fully explain the macroscopic development of a biological system. The mechanisms underlying plant color formation and control are species-specific. To date, few studies have been conducted on wheat grain color formation. Chen et al. (2020) elucidated the pathway regulating modifications to the major flavonoids in wheat grains. They analyzed the associations between metabolites and the whole genome in 182 common wheat accessions. Two candidate enzymes catalyzed the glycosylation and subsequent malonate acidification of various flavonoids, thereby establishing a modification pathway of the major flavonoids in wheat grains. Metabolomics and gene expression analysis revealed phenylpropane and flavonoid accumulation in wheat grains with various colors (Wang et al., 2020). To the best of our knowledge, no previous study has performed integrated metabolomic and transcriptomic analyses of the mechanisms controlling wheat grain color formation. Furthermore, most previous studies focused exclusively on color formation in the mature plant. Nevertheless, the process of color formation at the wheat grain-filling stage has not been reported. Therefore, the present study applied metabolomics and transcriptomics to clarify the mechanisms underlying wheat grain color formation at the filling stage. To this end, we examined the differences between colored (purple and blue) and common (white) wheat grains. We also evaluated differential metabolite and regulatory gene expression in colored wheat grains to elucidate the candidate genes and metabolic pathways controlling wheat grain color formation. We believe that the present study provides new perspectives on wheat color formation at the grain-filling stage and maturation.

## Materials and Methods

### Plant Materials and Cultivation Environment

Three varieties of fresh wheat, namely, Dianmai 20-1 (P, purple), Dianmai 20-8 (B, blue), and Dianmai 16 (W, white) were planted in early November 2019 at the Modern Agricultural Education and Research Base of Yunnan Agricultural University, Kunming City, Xundian County, Yunnan Province, China. The water management conditions were the same as those used during the field-planting period. Spikes of all three wheat lines were marked on the same day. Samples were taken at 7, 14, 21, 28, 35, and 42 days after flowering. Six middle spikelets were sampled per plant, frozen in liquid nitrogen, and stored in a −80°C Ultra-low temperature refrigerator for later use. At 28 days after anthesis when initial color formation occurred at the grain-filling stage, spikelets were selected for metabolite determination, transcriptome sequencing, and quantitative reverse transcription polymerase chain reaction (qRT-PCR) analysis; three biological replicates were used.

### Metabolomic Analysis

#### Sample Preparation and Metabolite Extraction

Wheat seeds were freeze-dried under vacuum in a lyophilizer (Scientz-100F; Ningbo Scientz Biotechnology Co. Ltd., Ningbo, Zhejiang, China) and crushed with zirconia beads in a blender (MM400; Retsch GmbH, Haan, Germany) at 30 Hz for 1.5 min. Then, 100 mg of powder was weighed, extracted with 0.6 mL of 70% (v/v) methanol, and stored in a refrigerator at 4°C overnight. The extract was then centrifuged at 10,000 × *g* at 4°C for 10 min, and the supernatant was passed through a 0.22-μm filter membrane and stored in an injection bottle for ultraperformance liquid chromatography-tandem mass spectrometry (UPLC-MS/MS) analysis.

#### Ultraperformance Liquid Chromatography-Tandem Mass Spectrometry Conditions

Chromatographic separation was conducted on an Agilent SB-C18 column (1.8 μm; 2.1 mm × 100 mm; Agilent Technologies, Santa Clara, CA, United States) at 40°C. The mobile phase consisted of solvent A [0.1% (v/v) formic acid in pure water] and solvent B (acetonitrile). The linear gradient program for elution was as follows: (a) from 0.00 to 9.00 min, phase B was in the range of 5–95% and maintained at 95% for 1 min; (b) from 10.00–11.10 min, the proportion of phase B was reduced to 5% and held for 14 min. The flow rate was 0.35 mL/min, and the injection volume was 4 μL. The effluent was alternately connected to an electrospray ionization-triple quadrupole-linear ion trap. The main conditions of the mass spectrometry were as follows: electrospray ionization temperature, 550°C; MS voltage, 5,500 V; curtain gas pressure, 30 psi; and collision-activated dissociation parameter, high. In triple quadruple pole (QQQ) mode, each ion pair was scanned according to the optimized declustering potential and collision energy (Chen et al., 2013).

#### Qualitative and Quantitative Metabolite Analyses

Qualitative metabolite analysis was performed using the in-house Metware database (Metware Biotechnology Co., Ltd., Wuhan, China) according to secondary spectral information. Isotopic signals, repeated signals containing K^+^, Na^+^, and NH4^+^ ions, and repeated signals of other high-MW debris ions were removed during the analysis. Metabolites were quantified by multiple reaction monitoring (MRM) analysis and QQQ-MS. During the instrumental analysis, a quality control (QC) sample was inserted after every tenth test and sample. Repeatability of the total ion flow detection method was determined by testing the spectra of various QC samples. Principal component (PCA) and orthogonal partial least squares discriminant analyses (OPLS-DA) were conducted on all metabolites to identify putative biomarkers. Metabolites with significantly different metabolism were selected as biomarkers based on FoldChange ≥ 2 and FoldChange ≤ 0.5.

### Transcriptome Sequencing and Data Analysis

#### RNA Extraction, Quantification, and Sequencing

Transcriptome sequencing comprises RNA extraction, detection, library construction, and computer sequencing. Total RNA was extracted from the seeds of the three wheat varieties. Agarose gel electrophoresis was used to assess the integrity of the RNA and detect any DNA contamination. RNA concentration was measured with a Qubit^®^ 2.0 fluorometer (Thermo Fisher Scientific, Waltham, MA, United States). RNA integrity was assessed with an Agilent 2100 Bioanalyzer (Agilent Technologies). RNA sequencing and assembly were performed by Metware Biotechnology Co., Ltd. Nine libraries consisting of three replicates of three grain samples were constructed. Transcriptome sequencing was performed on an Illumina HiSeq platform (Illumina, San Diego, CA, United States).

#### Quantitative Real-Time Polymerase Chain Reaction

To verify the reliability of the transcriptome sequencing data, all samples were subjected to qRT-PCR in three biological replicates. The ATP-dependent 26S proteasome regulatory subunit (26S, Primer sequence5′ -3′: Forward-GCTGGCTCGTTCAACTGATG, Reverse-GGACCAAGCGTTCTGATTACTC) was the internal reference gene. The primers (Supplementary Table 1) used in the qRT-PCR analysis were designed with Beacon Designer v. 7.9 (PREMIER Biosoft, Palo Alto, CA, United States). The 2^–ΔΔCt^ method was used to calculate the relative gene expression level (Livak and Schmittgen, 2001).

#### Transcriptome Data Analysis

A differential expression analysis was conducted on the various sample groups. The *P*-value was corrected by multiple hypothesis tests and the Benjamini–Hochberg method to obtain the false discovery rate (FDR). The thresholds of significantly different gene expression were | log2FoldChange| ≥ 1 and FDR < 0.05. Functional annotation and enrichment, new gene, alternative splicing, SNP, and InDel analyses were performed after screening differentially expressed genes (DEGs).

### Integrated Metabolomic and Transcriptomic Analyses

The transcriptome and metabolome data were normalized and statistically analyzed to establish the relationships between the genes and the metabolites implicated in wheat grain color development. PCA, Kyoto Encyclopedia of Genes and Genomes (KEGG) pathway and enrichment, correlation, two-way orthogonal partial least squares (O2PLS), and other analyses were also conducted. Interactive comparisons of metabolomic and transcriptomic data identified potential metabolites and their corresponding DEGs at the molecular and biochemical pathway levels.

## Results

### Widely Targeted Metabolomic Analysis of Three Types of Wheat Grain Color

**FIGURE 2:**
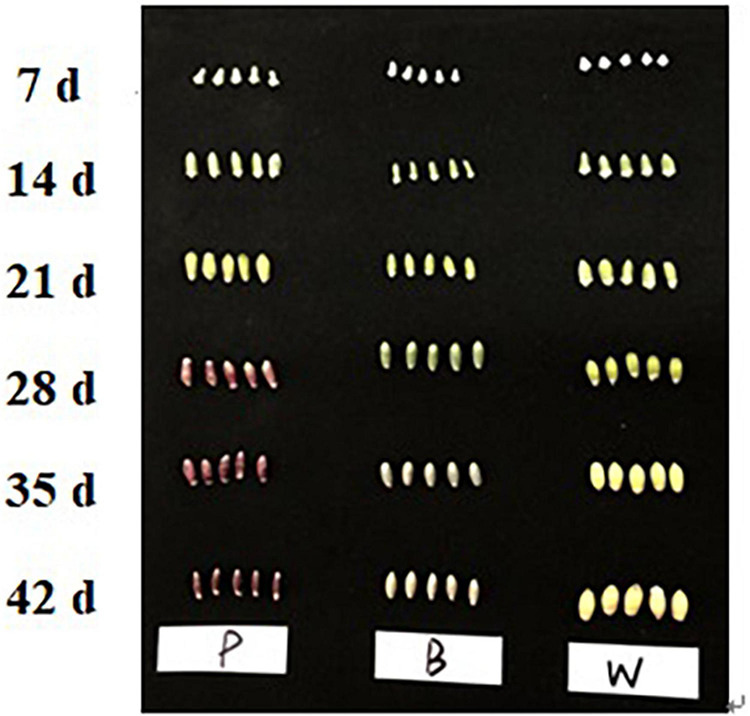
Three wheat seed varieties at different developmental stages.

**FIGURE 3:**
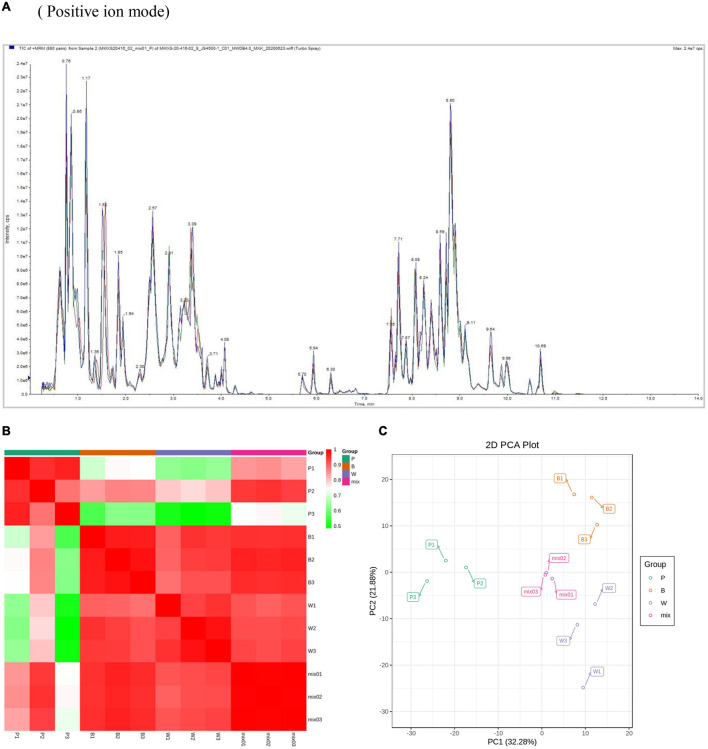
Overall qualitative and quantitative analyses of metabolomics data. **(A)** TIC overlap map of QC samples by MS detection. Abscissa represents retention time (min) of metabolite detection. Ordinate represents intensity of ion current (cps: count per second). **(B)** Pearson’s correlation coefficients among P (purple), B (blue), W (white), and QC samples (mixture). **(C)** PCA of P, B, W, and QC samples (mixture). *X*-axis represents the first principal component. *Y*-axis represents the second principal component.

In this experiment the grain colors of three materials Dianmai 20-1, Dianmai 20-8, and Dianmai 16 were purple, blue and white (amber), respectively. The grain shape of colored wheat P, B is oblong and white wheat is ovoid (Figure 2). Metabolites in the samples were detected by UPLC-MS/MS and qualitatively and quantitatively analyzed by MS. The total ion current (TIC) for the QC sample mixture and multi-peak diagram of MRM metabolite detection are shown in Supplementary Figures 1A,B. The MRM metabolite detection multi-peak diagram shows all substances detected in the samples. Of the 603 metabolites, the larger proportion was 111 lipids, 98 flavonoids (Supplementary Table 2), 78 amino acids and their derivatives, and 78 phenolic acids (Supplementary Table 3). Analysis of the TIC graphs (Figure 3A) plotted for MS detection and the various QC samples showed that the TIC metabolite detection curves had a high degree of overlap. Hence, retention times and peak intensities were equal among samples, and MS occurred at different times. Spot detection of the same samples demonstrated stable signals. Thus, metabolite extraction and detection were reliable. Pearson’s correlation coefficient r was used to evaluate biological repetition of samples within each group. As *r*^2^ approached unity, the stronger the correlation between two replicate samples (Figure 3B). A cluster heat map analysis was performed on all metabolites. The sample population cluster heat map showed that all three biological replicates of each species were clustered together (Supplementary Figure 1C). The PCA revealed the degree of metabolic variation between groups and among samples within the same group. P, B, W, and their combinations within the distribution of apparent dispersion showed that the metabolite levels differed among samples. P and B were clearly separated from W between PC1 and PC2 (Figure 3C). Therefore, the materials were sufficiently reproducible, suitable for use in the subsequent qualitative and quantitative analyses, and adequate to ensure the repeatability and reliability of the metabolomic data.

### Metabolic Differences Among Three Wheat Grain Colors

**FIGURE 4:**
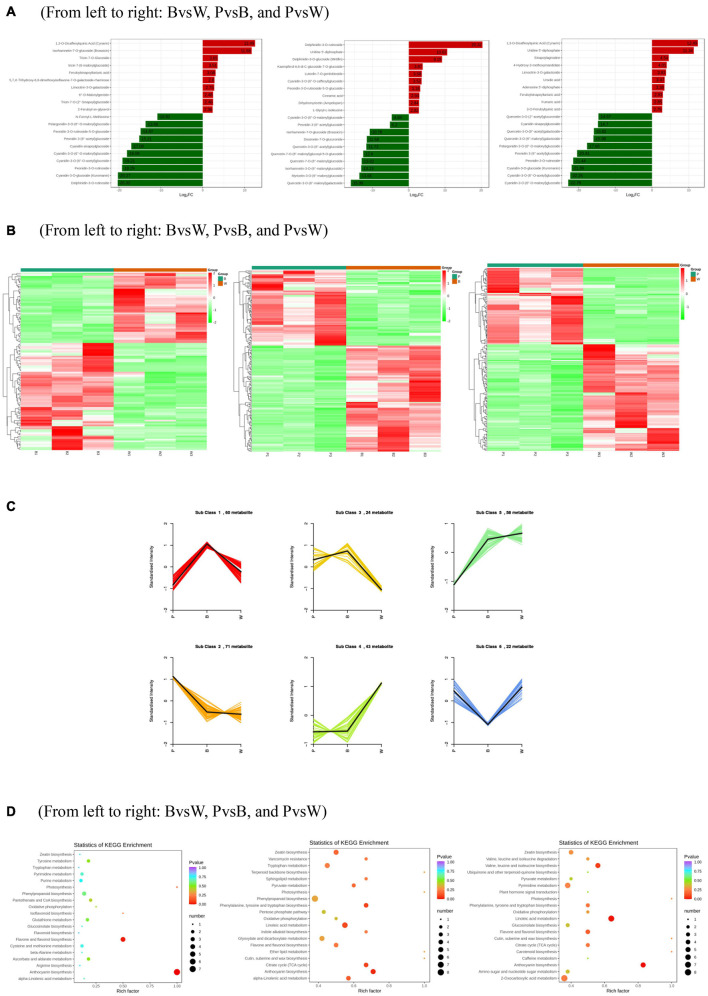
**(A)** Differentially expressed metabolite bar graph. Horizontal coordinates are log2FC of differential metabolites and vertical coordinates are differential metabolites. Red represents up-regulated differentially expressed metabolites, green represents down-regulated differentially expressed metabolites. **(B)** Differentially expressed metabolite clustering heat map. **(C)** *K* means analysis. **(D)** KEGG enrichment of differentially expressed metabolites. The horizontal coordinate indicates the Rich factor of each pathway, the vertical coordinate is the name of the pathway, and the color of the dot is the *P*-value, the redder it is, the more significant the enrichment. The size of the dots represents the number of differential metabolites enriched. **(A)** Horizontal coordinates are log2FC of differential metabolites and vertical coordinates are differential metabolites. Red represents up-regulated differentially expressed metabolites, green represents down-regulated differentially expressed metabolites. **(D)** The horizontal coordinate indicates the Rich factor of each pathway, the vertical coordinate is the name of the pathway, and the color of the dot is the *P*-value, the redder it is, the more significant the enrichment. The size of the dots represents the number of differential metabolites enriched.

Differential metabolism showed 23 metabolites in all three groups as well as 29, 48, and 30 unique metabolites in BvsW, PvsB, and PvsW, respectively. There were 111 metabolites that significantly differed in BvsW. Of these, the expression of 44 was upregulated and that of 67 was downregulated. There were 186 significantly different metabolites in PvsB. Of these, the expression of 109 was upregulated and that of 77 was downregulated. There were 175 significantly different metabolites in PvsW. Of these, the expression of 102 was upregulated and that of 73 was downregulated (Table 1). A PCA was performed on the samples, and significant separation of the first principal component was observed among groups (Supplementary Figure 2). The OPLS-DA (Supplementary Figure 3A) revealed that the first principal component was clearly separated among samples and that the model was reliable (Supplementary Figure 3B), and there were significant differences among metabolites in the S-plot of the OPLS-DA (Supplementary Figure 3C). Hence, there were substantial differences among the three grain colors in terms of their metabolite profiles. Based on the OPLS-DA results, metabolites with Fold Change ≥ 2 (or Fold Change ≤ 0.5) and variable importance in projection ≥ 1 were deemed significantly different. There was significant metabolite accumulation among the samples, and primarily, the flavonoids had changed (Figure 4A). The metabolites showed contrasting expression patterns among the differently colored wheat grains (Figure 4B). Flavonoid content was higher in colored wheat than in common wheat and was the highest in purple wheat (Supplementary Figure 3D). The *K* means analysis grouped metabolites with the same changing trend, revealing that the levels of most flavonoids were generally higher in purple wheat than in blue or common wheat. However, the lipid content was higher in common wheat than in colored wheat (Figure 4C). The KEGG analysis annotated, classified, and enriched the significantly different metabolites according to the pathways to which they belonged. Most of the significantly different metabolites were categorized into the metabolic pathway, followed by the specialized metabolite pathway. In general, the anthocyanin biosynthesis pathways were highly enriched. In addition, the flavonoid and flavonol biosynthesis pathways were highly enriched in the B and W groups, whereas the phenylalanine, tyrosine, and tryptophan biosynthesis pathways were highly enriched in the P and B groups. The linoleic acid metabolic pathway was highly enriched in the P and W groups (Figure 4D).

**TABLE 1:** Statistical classification of differentially expressed metabolites and genes.

Group	No. metabolites	Downregulated metabolites	Upregulated metabolites	No. DEGs	Downregulated DEGs	Upregulated DEGs
B_vs_W	111	67	44	6,062	3,017	3,045
P_vs_B	186	77	109	8,344	4,316	4,028
P_vs_W	175	73	102	10,476	5,397	5,079

### Transcriptomic Analysis of Three Types of Wheat Grain Color

**FIGURE 5:**
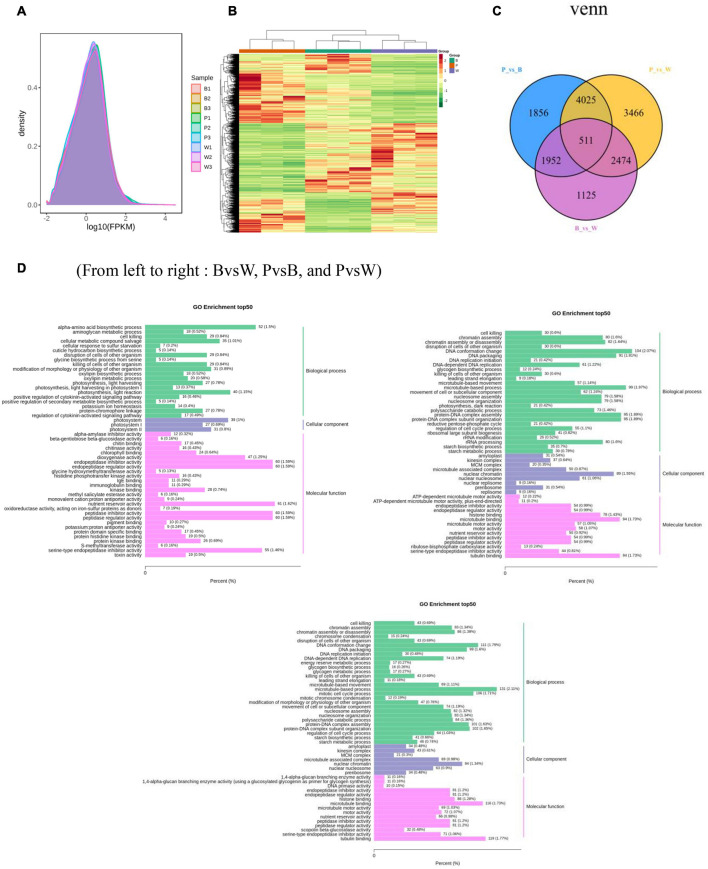
**(A)** Gene expression density distribution map. **(B)** Differential gene clustering heat map. **(C)** Differential gene Venn diagram. **(D)** Differential gene GO enrichment bar chart. **(A)** The curves of different colors in the figure represent different samples, the horizontal coordinates of the points on the curves indicate the logarithmic values of FPKM of the corresponding samples, and the vertical coordinates of the points indicate the probability density. **(B)** Horizontal coordinates indicate sample names and hierarchical clustering results, and vertical coordinates indicate differential genes and hierarchical clustering results. Red indicates high expression, green indicates low expression.

For the transcriptome sequencing analysis of the nine wheat samples, the sample correlation heat map (Supplementary Figure 4A) and PCA diagram (Supplementary Figure 4B) demonstrated suitable biological reproducibility within each wheat line and distinct differences among wheat lines. We generated 57.98 Gb of clean data after filtration. We obtained 6 Gb of high-quality reads per sample, and the proportion of Q30 bases was >93%. When the clean data were compared to those of the reference genome, the rate of correspondence was in the range of 82.85–91.71%. The gene expression density diagram for the samples indicated that the gene abundance in the nine samples had the same trend as that of the change in gene expression. Moreover, log FPKM was concentrated in the [−2, 2] interval and initially increased and decreased thereafter (Figure 5A). The detected genes were functionally annotated and the results were extracted. GO, KEGG, KOG, NR, Pfam, Swiss-Prot, and Tremble were annotated to 83,510, 76,228, 93,319, 106,835, 219,627, 75,389, and 107,372 genes, respectively. KEGG was enriched in 142 pathways.

### Analysis of Transcriptome Differences Among Three Wheat Grain Colors

DESeq2 was used to identify DEGs. In total, 15,409 DEGs were detected. The total number of DEGs (both upregulated and downregulated genes) per group is listed in Table 1. There are 6,062 DEGs in BvsW. Of these, 3,045 are upregulated and 3,017 are downregulated. 8,344 DEGs exist in PvsB, 4,028 are upregulated and 4,316 are downregulated. There are also 10,476 DEGs in PvsW, 5,079 are upregulated and 5,397 are downregulated. The overall distribution of the DEGs in both sample groups is shown in Figure 5B. Extraction of the centralized and standardized FPKM expression of the DEGs, analysis of hierarchical clusters, and cluster heat map plotting for each group revealed clear distinctions among the DEGs for the three wheat grain color groups. Thus, our sequencing data were reliable. There were 511 DEGs common to all three groups. However, Gene: TraesCS6A02G029800 was not detected in common wheat. There were 3,466 unique DEGs in PvsB, 1,125 unique DEGs in BvsW, and 1,856 unique DEGs in PvsW (Figure 5C). The pathway annotations of the DEGs to the KEGG database were analyzed, and it was determined that the primary metabolism and specialized metabolite pathways were the most prominent (Figure 5D). In the enrichment analysis of the DEGs, 50 GO terms with the lowest *q*-values were selected, and an enrichment column diagram was plotted. For BvsW, molecular function, biological process, and cellular component were 17.97, 11.61, and 2.49%, respectively. For PvsB, molecular function, biological process, and cellular component were 24.19, 5.87, and 13.36%, respectively. For PvsW, molecular function, biological process, and cellular component were 27.88, 5.09, and 14.67%, respectively. TF prediction identified 236 WRKY, 499 MYB, and 346 bHLH TFs.

### Quantitative Real-Time Polymerase Chain Reaction

**FIGURE 6:**
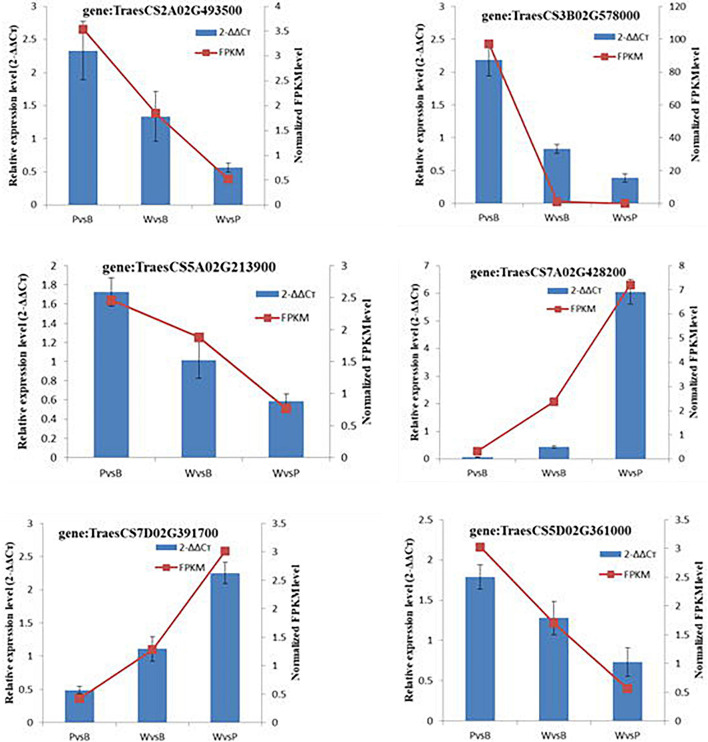
qRT-PCR validation of transcription levels of randomly selected DEGs.

The randomly selected DEGs were each subjected to fluorescence RT-qPCR for three times, and the normalized expression of each sample was analyzed with a 2^–ΔΔCt^ curve. There was good correlation between the sequencing results and the expression patterns detected by qRT-PCR. Hence, the transcriptome sequencing results were reliable (Figure 6).

### Combined Transcriptomic and Metabolomic Analyses Revealed the Mechanism by Which Wheat Grain Color Changes at the Grain-Filling Stage

**FIGURE 7:**
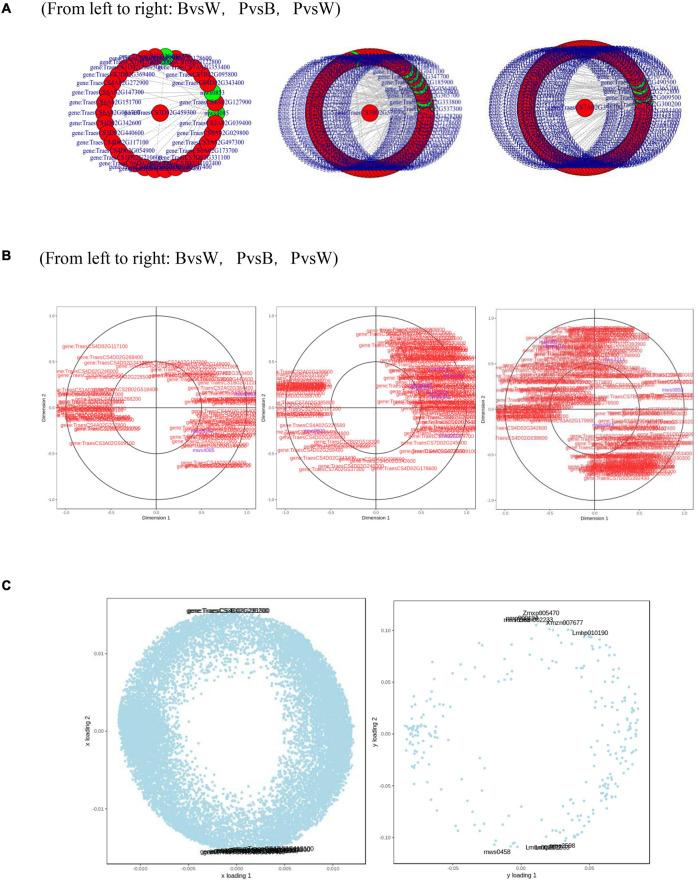
**(A)** Network diagram of correlations between metabolites and genes. **(B)** Canonical correlation analysis (CCA). **(C)** Left, transcriptome loading plot; right, metabolome loading plot. CYP73A, *trans*-cinnamate 4-monooxygenase; HCT, shikimate *O*-hydroxycinnamoyltransferase; CHS, chalcone synthase; CHI, chalcone isomerase; CYP75A, flavonoid 3′,5′-hydroxylase; CYP75B1, flavonoid 3′-monooxygenase; DFR, bifunctional dihydroflavonol 4-reductase; FLS, flavonol synthase; ANS, anthocyanidin synthase; F3H, naringenin 3-dioxygenase; ANR, anthocyanidin reductase.

Metabolome and transcriptome data were integrated to clarify the differences in color formation among wheat grain varieties. The PCA of the transcriptome and metabolome revealed that P accounted for most of the first principal component, while B comprised the majority of the second principal component. There were few differences between sample groups in terms of their transcriptomes and metabolomes. The most abundant pathways in all three groups included amino acid biosynthesis, cysteine and methionine metabolism, glutathione metabolism, arginine biosynthesis, arginine and proline metabolism, and flavonoid and flavonol biosynthesis. The KEGG enrichment analysis showed that the differentially expressed metabolites (DEMs) and DEGs related to color formation were strongly enriched in flavonoid and flavonol biosynthesis in PvsB and PvsW as well as cyanidin biosynthesis in BvsW. The DEMs with correlation coefficients >0.8 were used to plot a correlation coefficient clustering heat map indicating the highest proportion of flavonoids in all three groups (Supplementary Figure 4C). DEGs and DEMs with correlation coefficients >0.8 per pathway were used to plot a network diagram showing the correlations among metabolites and genes. The DEGs and DEMs formed a closely related network for phenylpropane compound biosynthesis (Figure 7A). The metabolites with strong correlations with the most genes in BvsW, PvsB, and PvsW were sinapyl alcohol, cinnamic acid, and ferulic acid, and the correlations were mainly positive.

**FIGURE 8:**
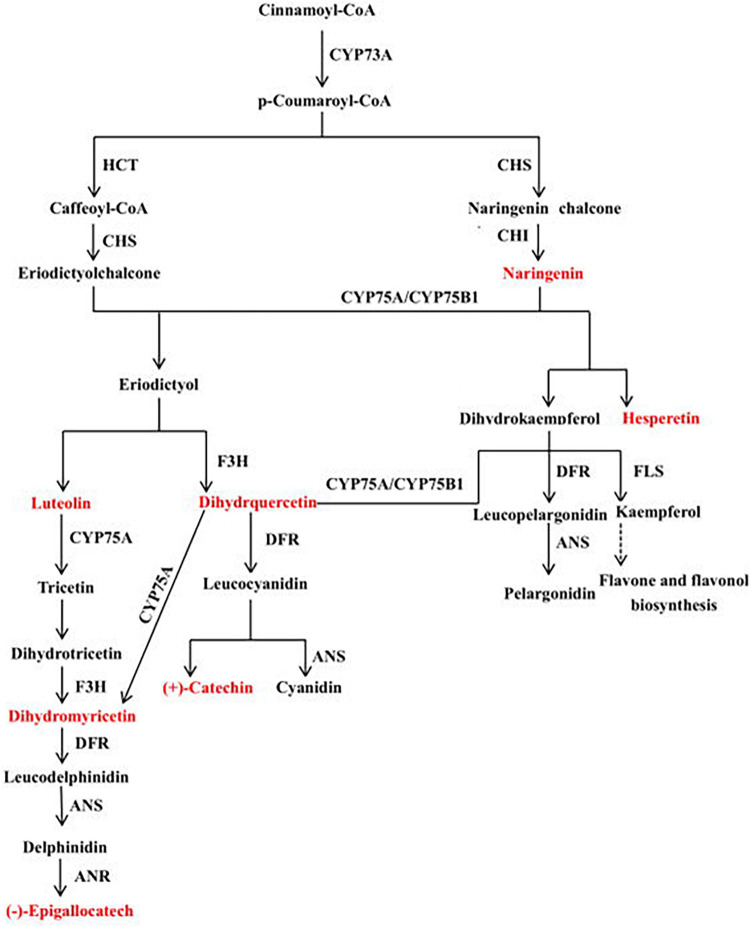
Mechanisms underlying flavonoid biosynthesis in wheat varieties with different grain colors. This pathway was constructed based on KEGG pathway and literature sources.

A canonical correlation analysis of phenylpropane compound biosynthesis showed that the DEGs and DEMs were highly correlated in all three groups (Figure 7B). An integrated O2PLS analysis of the transcriptome and metabolome datasets is shown in Figure 7C. Because of the transcription and metabolic group mutual influence, alterations in transcriptome data variables strongly affected the metabolomics. The top 10 genes with transcriptomes strongly influencing the metabolome and the top 10 metabolites substantially affecting the transcriptome are listed in Supplementary Table 4. Integrated metabolomic and transcriptomic analyses of the flavonoid biosynthesis pathway involved in the formation of wheat grain colors (Figure 8) revealed that under the phenylpropane biosynthesis pathway, flavonoids were biosynthesized from cinnamoyl-CoA and *p*-Coumaroyl-CoA through the action of CYP73A. For each wheat grain color, the metabolite type, level, and related enzymes were closely related. Among the genes related to flavonoid synthesis, Gene:TraesCS1B02G023600 was only present in colored wheat seeds, and the relative expression of Gene:TraesCS7A02G127600 in B, P, and W was 72.49, 36.61, and 32.49, respectively. These discrepancies resulted in different levels of pelargonidin, cyanidin, delphinidin and, by extension, different anthocyanin biosynthesis pathways among wheat grain types.

## Discussion

In the present study, the grains of the purple and blue wheat cultivars began to develop color at 28 days after anthesis. Hence, grain color regulation and formation began during the mid- to late grain-filling stage. A large number of studies have shown that the biosynthesis of phenylpropanes, flavonoids, isoflavones, flavonols, and anthocyanins is closely associated with plant color formation (Lou et al., 2014; Cho et al., 2016; Wang et al., 2017, 2018). The B ring of anthocyanin has different types and numbers of substituents, which determine the color hue and chromaticity of anthocyanin in specific tissues and cellular environments (Espley et al., 2007). Among the three wheat varieties tested here, 98 types of flavonoids were identified, accounting for 16.25% of the total metabolites. These 98 flavonoids included 37 flavonoids, 22 flavonols, 15 anthocyanins, 9 flavonoid carbon glycosides, 5 isoflavones, 4 dihydroflavonoids, 3 dihydroflavonols, and 3 flavanols. In grape hyacinth flowers, the diversion of the anthocyanin synthesis pathway probably accounts for the apricot flower decolorization. Delphinidin is responsible for the blue color change in grape hyacinth flowers. Competition between flavonol synthase and dihydroflavonol-4-reductase for the same substrate results in the disappearance of the blue pigment. In other words, the composition and content of anthocyanins affect the color change of grape hyacinth flowers (Lou et al., 2014). These previous findings are consistent with those of this study; herein, the levels of 46 flavonoids were higher in purple and blue wheat than in common wheat, and the levels of 14 flavonoids were higher in purple wheat than in the other varieties. Two types of delphinidins were detected in colored wheat, and their levels were higher than those in common wheat. The relative delphinidin-3-*O*-rutinoside content was higher in blue wheat, delphinidin might thus be a major source of pigmentation in blue wheat, as indicated by the above previous study. The relative contents of most anthocyanins were higher in purple and blue wheat than in common wheat, and the relative contents of most flavonols were higher in purple wheat than in blue wheat. Of the 23 differential metabolites identified in the three varieties of wheat, there were 16 types of flavonoids, and the levels of ten displayed the same trend, namely purple ≥ blue ≥ white wheat. Therefore, the mechanism of grain color formation in wheat is similar to that in other plants and is determined by the types of flavonoids present as well as the anthocyanin content and composition.

Sharply upregulated FcANS1 expression was revealed in the peel of a dark-colored fig during fruit ripening (Cao et al., 2016), whereas UFGT was identified as the critical gene for anthocyanin biosynthesis in grape and strawberry (Kobayashi et al., 2001; Griesser et al., 2008). Anthocyanidin-3-*O*-glucosyltransferase (3GT) is the last key enzyme in the anthocyanin biosynthetic pathway, which can catalyze unstable anthocyanidin into anthocyanin (Xin et al., 2011). Gene: TraesCS1B02G023600 regulates 3GT and is significantly associated with cypermethrin biosynthesis. In the present study, its expression was relatively upregulated in W vs. P and W vs. B. This is the same as brightly colored fruits commonly show a high gene expression of the key downstream enzymes of the anthocyanin biosynthetic pathway (Han et al., 2012). This gene might thus be pivotal in determining grain color differences among wheat varieties. In blue and common wheat, Gene: TraesCS7A02G127600 regulates caffeoyl-CoA-*O*-methyltransferase activity and is significantly associated with luteolin biosynthesis. The relative expression level of this wheat grain gene was not less than 20, and the order of expression level was blue > purple > common wheat. Therefore, this gene may be the principal factor determining the grain color differences among wheat varieties. The expression level of Gene: TRAESCS1D02G452300 in purple grain wheat was low in the phenylpropane biosynthesis pathway of purple wheat and Gene: TraesCS4B02G177300 expression level was extremely low in the anthocyanin biosynthesis pathways of common wheat. In isoflavone biosynthesis, the expression level of Gene: TraesCS3A02G458100 and Gene: TraesCS3D02G450900 was significantly lower in common wheat than in colored wheat. Hence, gene regulation alters flavonoid composition and content, which, in turn, causes differences in grain color among different wheat varieties.

## Conclusion

Variation in the types and content of flavonoids among the three wheat cultivars may account for the differences in their grain color. Ninety-seven flavonoids were detected in purple wheat, 90 were found in blue wheat, and 79 were identified in common wheat. The relative concentrations of 46 flavonoids were higher in purple and blue wheat than in common wheat. Moreover, the grain levels of pelargonidin, cyanidin, and delphinidin differed among the three wheat varieties as the expression of the genes regulating the biosynthesis of these pigments varied among the wheat cultivars. The flavonoid synthesis-related genes Gene:TraesCS1B02G023600 and Gene:TraesCS7A02G127600 may play a key role in determining the pigment formation of wheat seeds. The results of the present study might serve as an empirical basis for future investigations into the molecular and biochemical mechanisms underlying wheat grain color formation and guide plant geneticists and horticulturists in the breeding and development of novel wheat varieties.

## Data Availability Statement

The original contributions presented in the study are publicly available. This data can be found here: National Center for Biotechnology Information (NCBI) SRA database under accession number SRP336529.

## Author Contributions

LL: writing – original draft and methodology. ZK: conceptualization and writing- review and editing. XH: formal analysis and methodology. YeL: data curation and visualization. YoL: data curation and investigation. QW and JL: methodology and visualization. PZ and YG: formal analysis and investigation. PQ: supervision, project administration, and funding acquisition. All authors contributed to the article and approved the submitted version.

## Conflict of Interest

The authors declare that the research was conducted in the absence of any commercial or financial relationships that could be construed as a potential conflict of interest.

## Publisher’s Note

All claims expressed in this article are solely those of the authors and do not necessarily represent those of their affiliated organizations, or those of the publisher, the editors and the reviewers. Any product that may be evaluated in this article, or claim that may be made by its manufacturer, is not guaranteed or endorsed by the publisher.

## Supplementary Material

Supplementary Figure 1 **(A)** Total ion current of one quality control sample by mass spectrometry detection and **(B)** multi-peak detection plot of metabolites in the multiple reaction monitoring mode. The abscissa represents the retention time (min) of metabolite detection, and the ordinate represents the intensity of the ion current (cps: count per second). **(C)** The overall cluster diagram of the sample.

Supplementary Figure 2 Grouping principal component analysis diagram. The *x*-axis represents the first principal component and the *y*-axis represents the second principal component.

Supplementary Figure 3 **(A)** Scores opls-DA plot. **(B)** OPLS-DA verification plot. **(C)** OPLS-DA S-plot. **(D)** Differences metabolites clustering heat maps.

Supplementary Figure 4 **(A)** Sample correlation heat map. **(B)** Gene principal component analysis diagram. **(C)** Correlation coefficient clustering heat maps.

## Abbreviations

ANR: anthocyanidin reductase
ANS: anthocyanidin synthase
bHLH: basic helix–loop–helix
CHI: chalcone isomerase
CHS: chalcone synthase
CYP73A: *trans*-cinnamate-4-monooxygenase
CYP75A: flavonoid-3′, 5′ -hydroxylase
CYP75B1: flavonoid-3′ -monooxygenase
DEG: differentially expressed gene
DEM: differentially expressed metabolite
DFR: bifunctional dihydroflavonol-4-reductase
F3H: naringenin-3-dioxygenase
FDR: false discovery rate
FPKM: fragments per kilobase per million mapped reads
GO: gene ontology
HCT: shikimate-*O*-hydroxycinnamoyl transferase
KEGG: Kyoto Encyclopedia of Genes and Genomes
MRM: multiple reaction monitoring
MYB: myeloblastosis
O2PLS: two-way orthogonal partial least squares
OPLS-DA: orthogonal partial least squares discriminant analysis
PCA: principal component analysis
QC: quality control
QQQ: triple quadrupole
qRT-PCR: quantitative real-time polymerase chain reaction
TF: transcription factor
TIC: total ion current
UPLC-MS/MS: ultraperformance liquid chromatography-tandem mass spectrometry
WDR: WD40 repeat protein.
