# Supplementary material for: Transcriptomics Integrated With Widely Targeted Metabolomics Reveals the Mechanism Underlying Grain Color Formation in Wheat at the Grain-Filling Stage
Source: Front Plant Sci. 2021 Oct 14;12:757750. doi: 10.3389/fpls.2021.757750 (PMC8551455; doi:10.3389/fpls.2021.757750)
Supplement: Supplementary Figure 1 — (A) Total ion current of one quality control sample by mass spectrometry detection and (B) multi-peak detection plot of metabolites in the multiple reaction monitoring mode. The abscissa represents the retention time (min) of metabolite detection, and the ordinate represents the intensity of the ion current (cps: count per second). (C) The overall cluster diagram of the sample. [file Data_Sheet_1.zip › Supplementary Table 2.docx]

| **Supplementary Table 2. 98 kinds of flavonoids** | | | | | | |
| --- | --- | --- | --- | --- | --- | --- |
| No. | Compounds | Class | Relative content | | |  |
|  |  |  | P | B | W |  |
| 1 | Hesperetin-5-O-glucoside | Dihydroflavonol | 374400.00 | 37488.67 | 31260.33 |  |
| 2 | Cyanidin-3-O-glucoside (Kuromanin) | Anthocyanins | 34930000.00 | 11346933.33 | 9.00 |  |
| 3 | Cyanidin-3-O-(6''-O-acetyl)glucoside | Anthocyanins | 45024666.67 | 5459800.00 | 9.00 |  |
| 4 | Peonidin 3-(6''-acetyl)glucoside | Anthocyanins | 12555933.33 | 341168.33 | 9.00 |  |
| 5 | Pelargonidin-3-O-(6''-O-malonyl)glucoside | Anthocyanins | 1887233.33 | 116808.33 | 9.00 |  |
| 6 | Cyanidin-3-O-(6''-O-malonyl)glucoside | Anthocyanins | 64913333.33 | 2515793.33 | 9.00 |  |
| 7 | Cyanidin-3-O-rutinoside (Keracyanin) | Anthocyanins | 33555333.33 | 25084333.33 | 409200.00 |  |
| 8 | Peonidin-3-O-rutinoside | Anthocyanins | 25558666.67 | 5777633.33 | 9.00 |  |
| 9 | Cyanidin-3-O-(2''-O-glucosyl)glucoside | Anthocyanins | 290256.67 | 777673.33 | 32523.00 |  |
| 10 | Petunidin-3-O-(6''-O-p-Coumaroyl)glucoside | Anthocyanins | 1063716.67 | 1263790.00 | 352510.00 |  |
| 11 | Cyanidin-sinapoylglucoside | Anthocyanins | 239896.67 | 1243296.67 | 9.00 |  |
| 12 | Peonidin-3-O-rutinoside-5-O-glucoside | Anthocyanins | 29786.33 | 269613.33 | 9.00 |  |
| 13 | Luteolin (5,7,3',4'-Tetrahydroxyflavone) | Flavonoid | 2161.17 | 20286.93 | 259.76 |  |
| No. | Compounds | Class | Relative content | | |  |
|  |  |  | P | B | W |  |
| 14 | Diosmetin (5,7,3'-Trihydroxy-4'-methoxyflavone) | Flavonoid | 756956.67 | 386256.67 | 168953.33 |  |
| 15 | 5,7-Dihydroxy-3',4',5'-trimethoxyflavone | Flavonoid | 10520.23 | 9414.23 | 7794.23 |  |
| 16 | Syringetin | Flavonoid | 28175.33 | 68404.00 | 38253.80 |  |
| 17 | Nobiletin (5,6,7,8,3',4'-Hexamethoxyflavone) | Flavonoid | 39805.00 | 30730.87 | 14152.00 |  |
| 18 | Naringenin-4'-O-glucoside | Flavonoid | 26008.67 | 8305.13 | 3426.43 |  |
| 19 | Luteolin-7-O-glucoside (Cynaroside) | Flavonoid | 223546.67 | 227370.00 | 14544.00 |  |
| 20 | Aromadendrin-7-O-glucoside | Flavonoid | 1566333.33 | 902423.33 | 729423.33 |  |
| 21 | Chrysoeriol-7-O-glucoside | Flavonoid | 327373.33 | 23719.67 | 18492.67 |  |
| 22 | Chrysoeriol-8-C-glucoside (Scoparin)* | Flavonoid | 181033.00 | 23964.33 | 5993.67 |  |
| 23 | Tricin-4'-O-syringyl alcohol | Flavonoid | 31887.33 | 24046.63 | 23942.00 |  |
| 24 | Apigenin-7-O-rutinoside (Isorhoifolin)* | Flavonoid | 306940.00 | 351713.33 | 296926.67 |  |
| 25 | Apigenin-6,8-di-C-glucoside | Flavonoid | 263456.67 | 311730.00 | 139263.00 |  |
| 26 | Diosmetin-7-O-rutinoside (Diosmin) | Flavonoid | 2013533.33 | 733710.00 | 678783.33 |  |
| 27 | Luteolin-7-O-gentiobioside | Flavonoid | 142086.67 | 1692033.33 | 294993.33 |  |
| No. | Compounds | Class | Relative content | | |  |
|  |  |  | P | B | W |  |
| 28 | Isorhamnetin-3-O-rutinoside (Narcissin) | Flavonoid | 1082773.33 | 1203683.33 | 337580.00 |  |
| 29 | chrysoeriol-7-O-diglucoside | Flavonoid | 183053.33 | 308390.00 | 94624.67 |  |
| 30 | Chrysoeriol-6-C-glucoside-4'-O-glucoside* | Flavonoid | 159964.67 | 18582.67 | 6278.07 |  |
| 31 | Tricin-5,7-diglucoside | Flavonoid | 128164.00 | 166276.67 | 72144.67 |  |
| 32 | Tangeretin | Flavonols | 18460.80 | 12655.93 | 6317.83 |  |
| 33 | Quercetin-3-O-arabinoside (Guaijaverin) | Flavonols | 26090.33 | 4064.33 | 2174.07 |  |
| 34 | Kaempferol-3-O-glucuronide | Flavonols | 32471.00 | 8412.50 | 9.00 |  |
| 35 | Quercetin-3-O-glucoside (Isoquercitrin)* | Flavonols | 617163.33 | 48400.33 | 34427.33 |  |
| 36 | Kaempferol-3-O-(6''-acetyl)glucoside | Flavonols | 74569.33 | 10183.43 | 9431.07 |  |
| 37 | Quercetin-3-O-(6''-acetyl)galactoside | Flavonols | 519216.67 | 21967.00 | 9.00 |  |
| 38 | Quercetin-3-O-(2''-acetyl)glucuronide | Flavonols | 218753.33 | 15495.33 | 9.00 |  |
| 39 | Isorhamnetin-3-O-(6''-acetylglucoside) | Flavonols | 430863.33 | 37166.00 | 4527.63 |  |
| 40 | Kaempferol-3-O-neohesperidoside-7-O-glucoside | Flavonols | 22681.00 | 27947.33 | 20745.67 |  |
| 41 | Luteolin-6-C-glucoside (Isoorientin) | Flavonoid carbonoside | 170539.67 | 34184.00 | 28058.33 |  |
| No. | Compounds | Class | Relative content | | |  |
|  |  |  | P | B | W |  |
| 42 | Luteolin-8-C-glucoside-6-C-arabinoside | Flavonoid carbonoside | 144930.00 | 146683.33 | 44642.33 |  |
| 43 | Vitexin-2''-O-glucoside | Flavonoid carbonoside | 41720.00 | 43650.33 | 18892.67 |  |
| 44 | Chrysoeriol-6,8-di-C-glucoside* | Flavonoid carbonoside | 172284.67 | 19626.00 | 3998.67 |  |
| 45 | Apigenin-6-C-glucoside-7-O-(6''-sinapoyl)glucoside | Flavonoid carbonoside | 51636.67 | 61484.00 | 46207.33 |  |
| 46 | 5,7,4'-Trihydroxyisoflavone-7-O-galactoside-rhamnose* | Isoflavones | 334763.33 | 364253.33 | 305960.00 |  |
| 47 | Naringenin (5,7,4'-Trihydroxyflavanone) | Dihydroflavone | 9795.70 | 18800.40 | 17629.33 |  |
| 48 | Hesperetin | Dihydroflavone | 5118.30 | 13263.97 | 14575.00 |  |
| 49 | Naringenin-7-O-Rutinoside(Narirutin) | Dihydroflavone | 46342.33 | 31948.67 | 47565.67 |  |
| 50 | Hesperetin-7-O-rutinoside (Hesperidin) | Dihydroflavone | 158276.67 | 66753.00 | 88789.67 |  |
| 51 | Dihydroquercetin(Taxifolin) | Dihydroflavonol | 13786.90 | 16776.93 | 16722.33 |  |
| 52 | Dihydromyricetin (Ampelopsin) | Dihydroflavonol | 4582.33 | 32729.00 | 22386.00 |  |
| 53 | Delphinidin-3-O-glucoside (Mirtillin) | Anthocyanins | 6381.37 | 3726933.33 | 12227.73 |  |
| 54 | Cyanidin-3-O-(6''-O-caffeoyl)glucoside | Anthocyanins | 129420.00 | 1484566.67 | 297260.00 |  |
| 55 | Delphinidin-3-O-rutinoside | Anthocyanins | 9.00 | 11801600.00 | 9.00 |  |
| No. | Compounds | Class | Relative content | | |  |
|  |  |  | P | B | W |  |
| 56 | Malvidin-3-O-(6''-O-p-coumaroyl)glucoside | Anthocyanins | 448060.00 | 507730.00 | 1482890.00 |  |
| 57 | 4',5,7-Trihydroxy-3',6-dimethoxyflavone (Jaceosidin)* | Flavonoid | 155087.33 | 46256.67 | 144106.67 |  |
| 58 | Tricin (5,7,4'-Trihydroxy-3',5'-dimethoxyflavone)* | Flavonoid | 451103.33 | 134679.33 | 422816.67 |  |
| 59 | Diosmetin-7-O-glucuronide | Flavonoid | 27554.00 | 9.00 | 9.00 |  |
| 60 | Tricin-7-O-Glucoside | Flavonoid | 264186.67 | 37159.00 | 464996.67 |  |
| 61 | Mearnsetin-3-O-glucoside | Flavonoid | 57937.00 | 63526.67 | 107878.00 |  |
| 62 | Limocitrin-3-O-galactoside | Flavonoid | 6364.03 | 13438.90 | 90466.33 |  |
| 63 | Salcolin A* | Flavonoid | 67304.00 | 91794.00 | 86852.33 |  |
| 64 | Salcolin B* | Flavonoid | 70085.00 | 98084.00 | 81389.00 |  |
| 65 | Chrysoeriol-7-O-(6''-malonyl)glucoside | Flavonoid | 107730.67 | 9539.43 | 16530.33 |  |
| 66 | Isorhamnetin-3-O-(6''-malonylglucoside) | Flavonoid | 80685.33 | 9.00 | 9.00 |  |
| 67 | tricin-7-(6-malonylglucoside) | Flavonoid | 178520.00 | 29908.67 | 341440.00 |  |
| 68 | Kaempferol-3-O-rutinoside(Nicotiflorin) | Flavonoid | 347903.33 | 187436.67 | 375766.67 |  |
| 69 | Tricin-7-O-rutinoside | Flavonoid | 450653.33 | 487210.00 | 1544250.00 |  |
| No. | Compounds | Class | Relative content | | |  |
|  |  |  | P | B | W |  |
| 70 | Tricin-4'-O-(β-guaiacylglycerol)ether-7-O-glucoside | Flavonoid | 2402.13 | 2449.00 | 6217.60 |  |
| 71 | Tricin-7-O-(2''-Sinapoyl)glucoside | Flavonoid | 2750.57 | 1290.00 | 7071.90 |  |
| 72 | Chrysoeriol-7-O-rutinoside-5-O-glucoside | Flavonoid | 96857.33 | 259810.00 | 119322.00 |  |
| 73 | Kaempferol-6,8-di-C-glucoside-7-O-glucoside | Flavonoid | 8584.53 | 124913.33 | 11958.27 |  |
| 74 | Syringetin-3-O-rutinoside-7-O-glucoside | Flavonoid | 87780.00 | 143330.00 | 132993.33 |  |
| 75 | Isorhamnetin | Flavonols | 5390.60 | 4994.10 | 1742.03 |  |
| 76 | Quercetin-3-O-galactoside (Hyperin)* | Flavonols | 782066.67 | 65529.00 | 44987.67 |  |
| 77 | Isorhamnetin-7-O-glucoside (Brassicin) | Flavonols | 15859.00 | 9.00 | 29604.33 |  |
| 78 | Myricetin-3-O-galactoside | Flavonols | 28911.33 | 23667.33 | 83578.00 |  |
| 79 | Quercetin-3-O-(6''-acetyl)glucoside | Flavonols | 30300.33 | 9.00 | 9.00 |  |
| 80 | Quercetin-3-O-(6''-malonyl)galactoside* | Flavonols | 580856.67 | 9.00 | 9.00 |  |
| 81 | Quercetin-7-O-(6''-malonyl)glucoside* | Flavonols | 74913.67 | 9.00 | 9.00 |  |
| 82 | Myricetin-3-O-(6''-malony)glucoside | Flavonols | 115648.67 | 9.00 | 9.00 |  |
| 83 | Quercetin-3-O-rutinoside (Rutin) | Flavonols | 1839733.33 | 753736.67 | 777113.33 |  |
| No. | Compounds | Class | Relative content | | |  |
|  |  |  | P | B | W |  |
| 84 | Isorhamnetin-3-O-glucuronide-7-O-rhamnoside | Flavonols | 405760.00 | 460350.00 | 1311993.33 |  |
| 85 | Quercetin-7-O-(6''-malonyl)glucosyl-5-O-glucoside | Flavonols | 55934.67 | 9.00 | 9.00 |  |
| 86 | Quercetin-7-O-rutinoside-4'-O-glucoside | Flavonols | 19670.00 | 39067.67 | 51367.67 |  |
| 87 | Isorhamnetin-3-O-rutinoside-4'-O-glucoside | Flavonols | 48527.67 | 99637.67 | 98544.67 |  |
| 88 | Apigenin-6-C-glucoside (Isovitexin) | Flavonoid carbonoside | 124313.33 | 39020.33 | 60849.00 |  |
| 89 | Schaftoside | Flavonoid carbonoside | 28166.00 | 13752.83 | 23630.67 |  |
| 90 | Isoschaftoside | Flavonoid carbonoside | 4800300.00 | 2668733.33 | 4391633.33 |  |
| 91 | Apigenin-6-C-glucoside-7-O-(6''-feruloyl)glucoside | Flavonoid carbonoside | 133743.33 | 326946.67 | 187506.67 |  |
| 92 | Catechin | Flavanols | 192913.33 | 401876.67 | 525473.33 |  |
| 93 | Epigallocatechin | Flavanols | 166013.33 | 530596.67 | 288733.33 |  |
| 94 | Gallocatechin-Gallocatechin | Flavanols | 92758.00 | 50904.67 | 70429.00 |  |
| 95 | 5,7,4'-Trihydroxyisoflavone-7-O-galactoside | Isoflavones | 22256.33 | 1432.80 | 6738.37 |  |
| 96 | 6''-O-Malonylgenistin | Isoflavones | 11008.60 | 2895.30 | 16202.67 |  |
| 97 | 5,7,4'-Trihydroxy-6,8-dimethoxyisoflavone-7-O-galactoside-rhamnose | Isoflavones | 12539.27 | 5370.00 | 37336.67 |  |
| No. | Compounds | Class | Relative content | | |  |
|  |  |  | P | B | W |  |
| 98 | 5,7,4'-Trihydroxy-6,8-dimethoxyisoflavone-7-O-galactoside-glucose-rhamnose | Isoflavones | 148623.33 | 208263.33 | 149200.00 |  |

Note: Blue and purple wheat are higher than white wheat in the top 46 flavonoids
