# Supplementary material for: Transcriptomics Integrated With Widely Targeted Metabolomics Reveals the Mechanism Underlying Grain Color Formation in Wheat at the Grain-Filling Stage
Source: Front Plant Sci. 2021 Oct 14;12:757750. doi: 10.3389/fpls.2021.757750 (PMC8551455; doi:10.3389/fpls.2021.757750)
Supplement: Supplementary Figure 1 — (A) Total ion current of one quality control sample by mass spectrometry detection and (B) multi-peak detection plot of metabolites in the multiple reaction monitoring mode. The abscissa represents the retention time (min) of metabolite detection, and the ordinate represents the intensity of the ion current (cps: count per second). (C) The overall cluster diagram of the sample. [file Data_Sheet_1.zip › Supplementary Table 4.docx]

| **Supplementary Table 4.The top 10 genes that the transcriptome has a greater impact on the metabolome and the top 10 metabolites that have a greater impact on the transcriptome** | | |
| --- | --- | --- |
| ID | compounds | length |
| gene:TraesCS3B02G235200 | | 0.015614 |
| gene:TraesCS6A02G188600 | | 0.015597 |
| gene:TraesCS6A02G021300 | | 0.015583 |
| gene:TraesCS1B02G113100 | | 0.015566 |
| gene:TraesCS1D02G267400 | | 0.015565 |
| gene:TraesCS4D02G281500 | | 0.015547 |
| gene:TraesCS1B02G024300 | | 0.015514 |
| gene:TraesCS1D02G443300 | | 0.015495 |
| gene:TraesCS6A02G174200 | | 0.015491 |
| gene:TraesCS6A02G170900 | | 0.015483 |
| Zmxp005470 | tricin-7-(6-malonylglucoside) | 0.117992 |
| mws0458 | Vanillin | 0.114911 |
| pme2598 | 3,4-Dihydroxybenzeneacetic acid | 0.112078 |
| pmp000194 | 6''-O-Malonylgenistin | 0.112068 |
| Xmzn007677 | Ergotamine | 0.112051 |
| Lmtp001633 | Peonidin-3-O-rutinoside-5-O-glucoside | 0.111909 |
| Lmtn002233 | Androsin | 0.110982 |
| Lmbn002072 | 2-Propylsuccinic acid | 0.110705 |
| mws0983 | N-Oleoylethanolamine | 0.110224 |
| Lmhp010190 | LysoPC 18:1(2n isomer) | 0.109945 |
