# Supplementary figures and images for: Transcriptomics Integrated With Widely Targeted Metabolomics Reveals the Mechanism Underlying Grain Color Formation in Wheat at the Grain-Filling Stage
Source: Front Plant Sci. 2021 Oct 14;12:757750. doi: 10.3389/fpls.2021.757750 (PMC8551455; doi:10.3389/fpls.2021.757750)

**Supplementary Figure 1.**

**(A)** ( Positive ion mode)


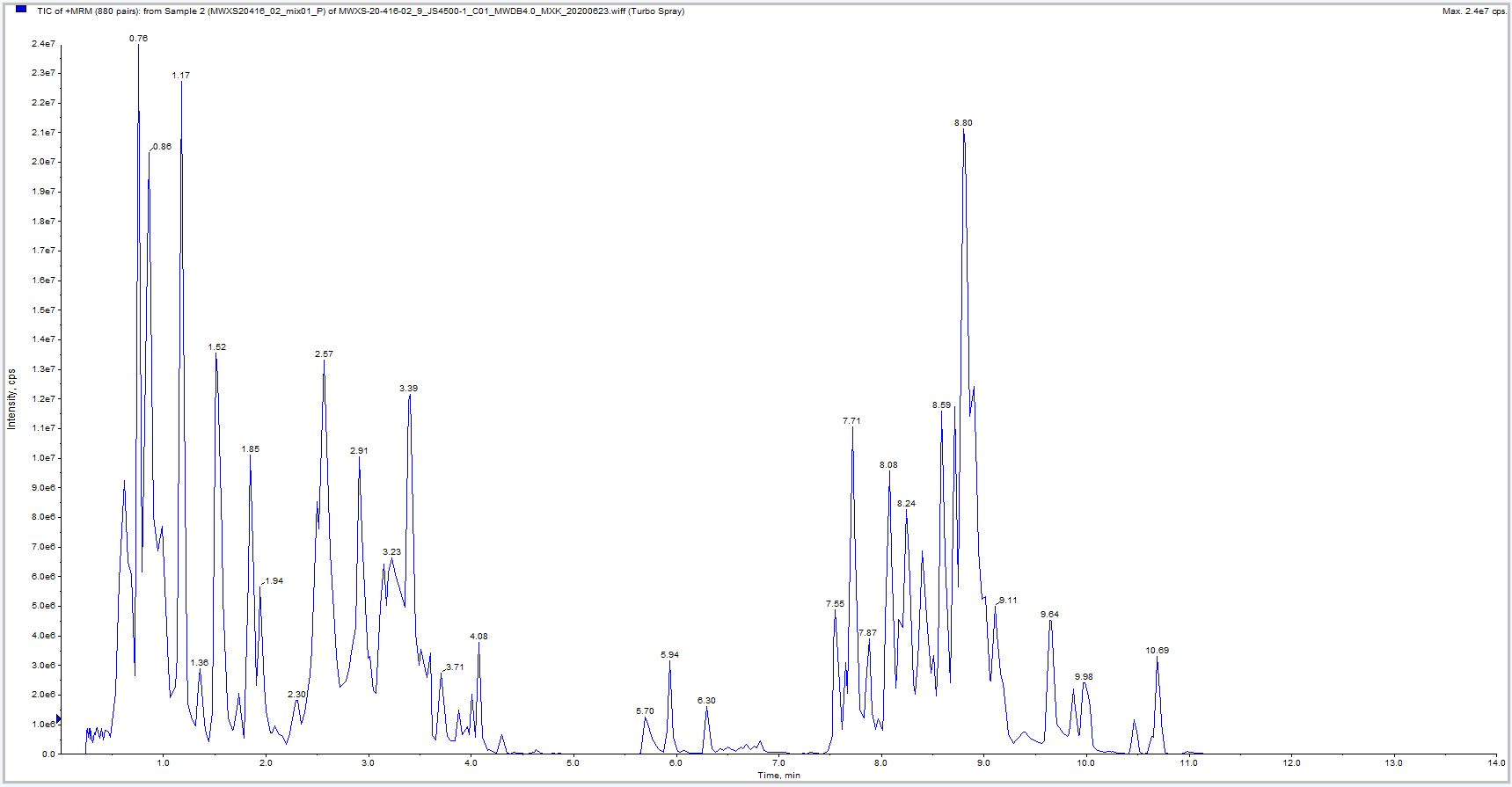


**(B)** ( Positive ion mode)


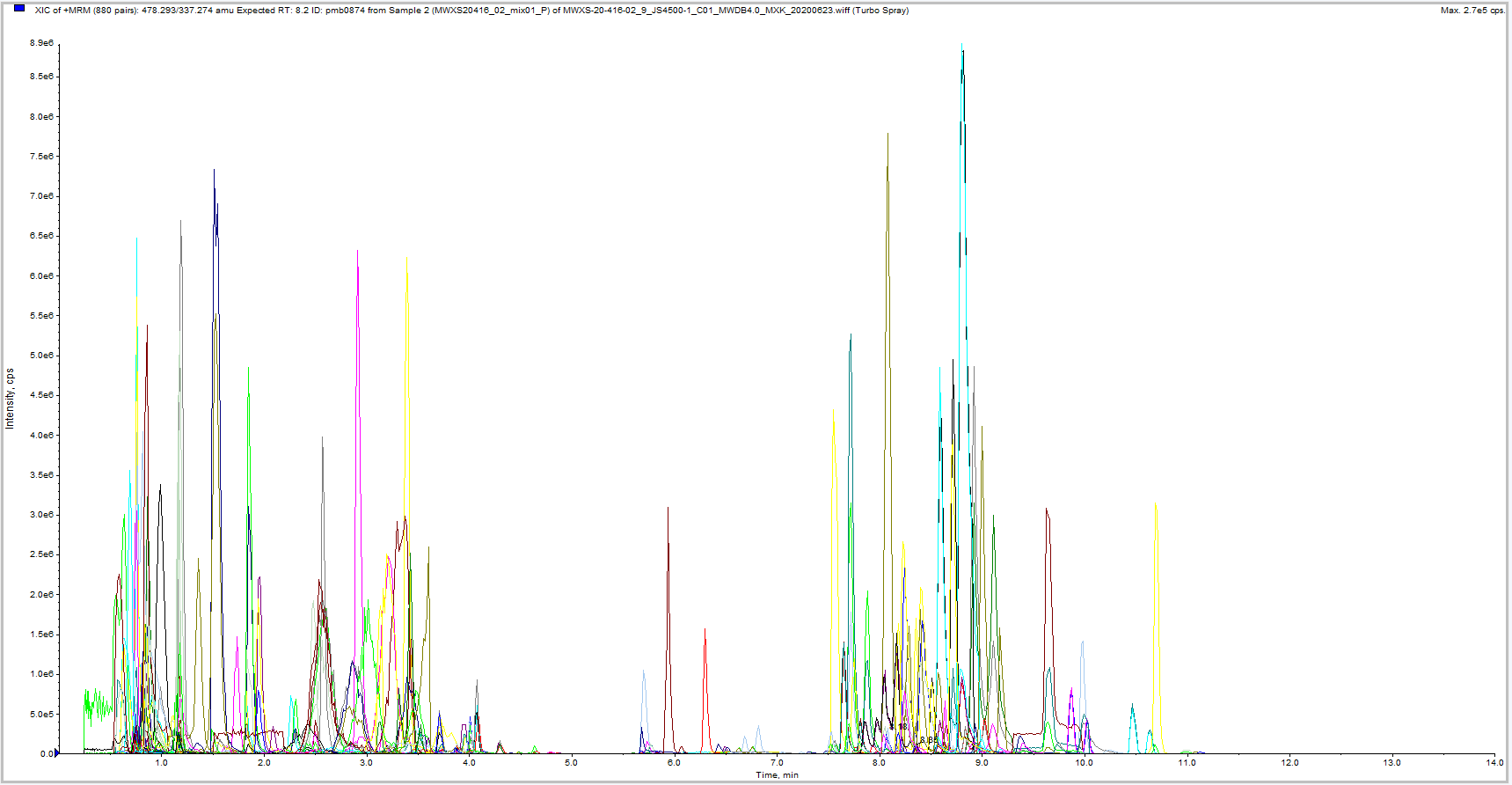


**(C)**


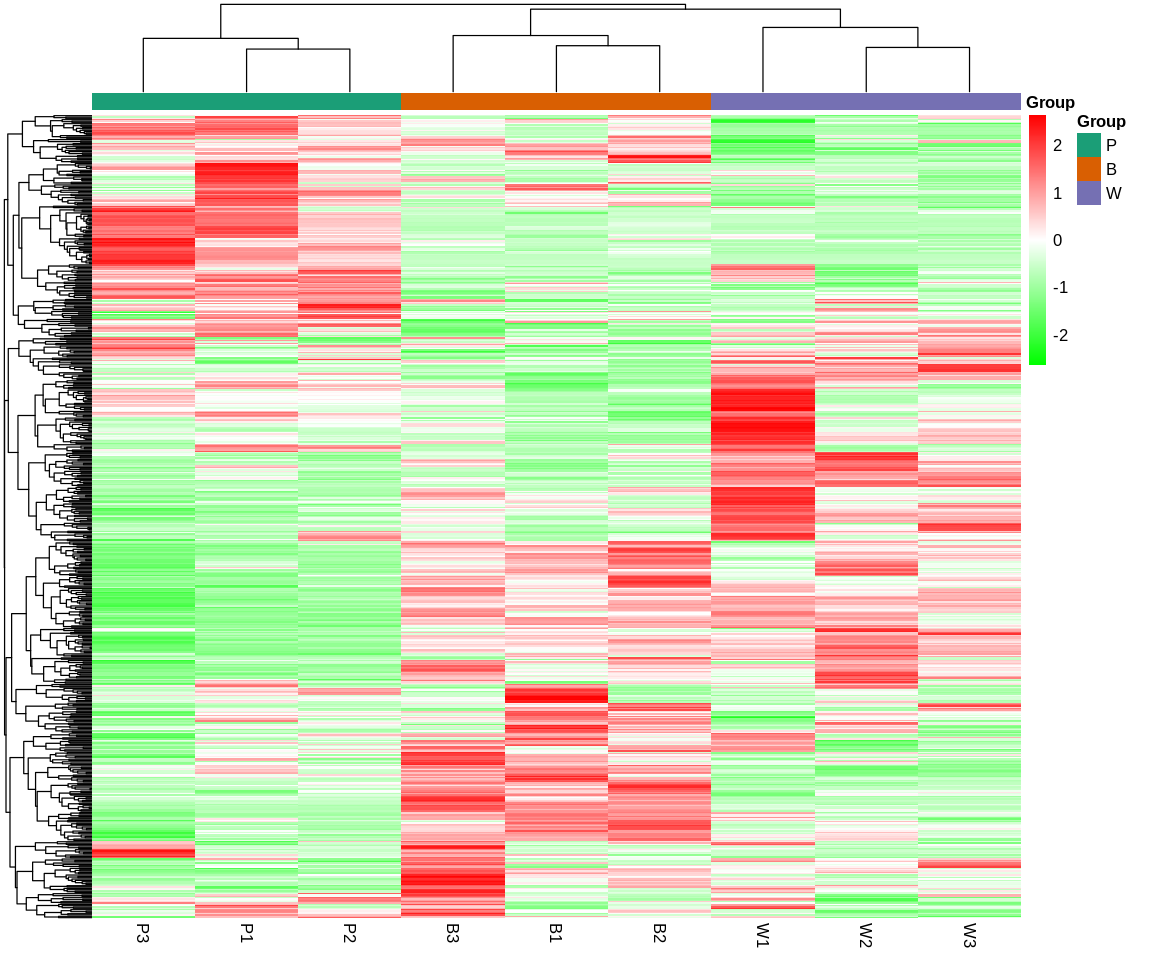

Supplement: Supplementary Figure 1 — (A) Total ion current of one quality control sample by mass spectrometry detection and (B) multi-peak detection plot of metabolites in the multiple reaction monitoring mode. The abscissa represents the retention time (min) of metabolite detection, and the ordinate represents the intensity of the ion current (cps: count per second). (C) The overall cluster diagram of the sample. [file Data_Sheet_1.zip › Supplementary Figure 1.docx]

**Supplementary Figure 2.**

(From left to right: BvsW, PvsB, and PvsW)


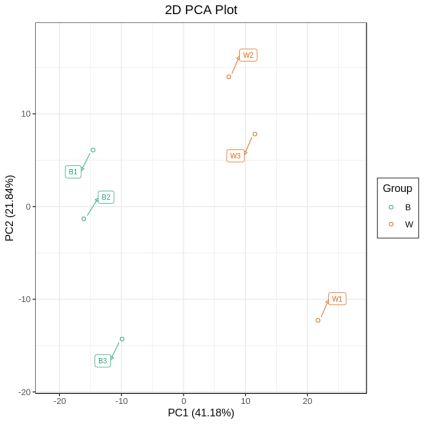
**
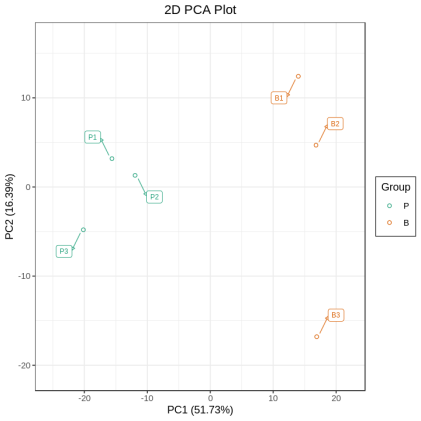
**
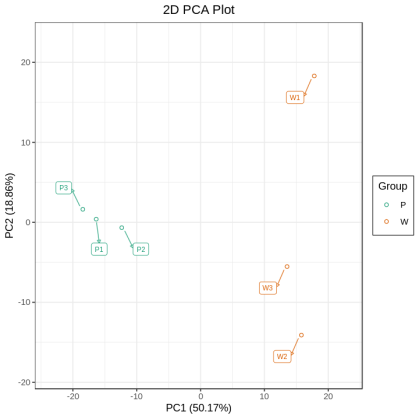

Supplement: Supplementary Figure 1 — (A) Total ion current of one quality control sample by mass spectrometry detection and (B) multi-peak detection plot of metabolites in the multiple reaction monitoring mode. The abscissa represents the retention time (min) of metabolite detection, and the ordinate represents the intensity of the ion current (cps: count per second). (C) The overall cluster diagram of the sample. [file Data_Sheet_1.zip › Supplementary Figure 2.docx]

**Supplementary Figure 3.**

**(A)** (From left to right: BvsW, PvsB, and PvsW)


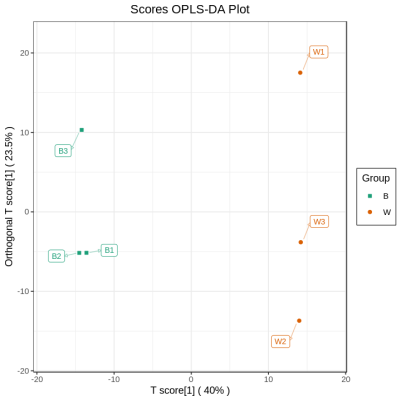

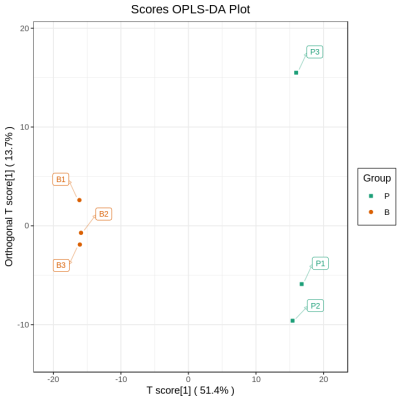

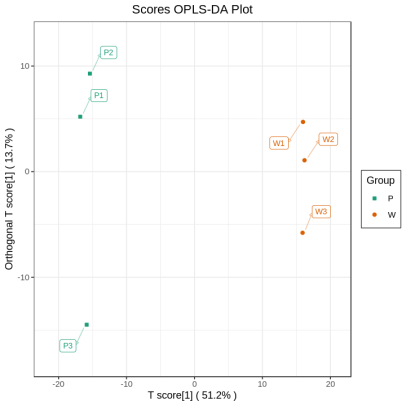


**(B)**


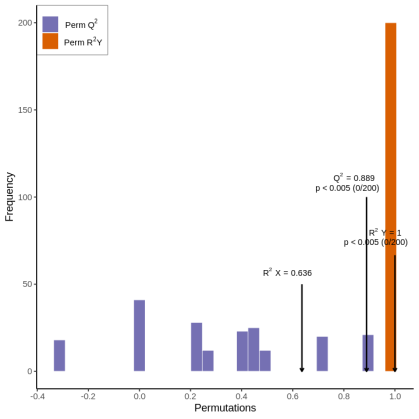

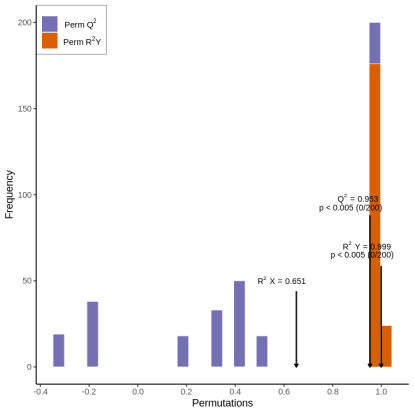

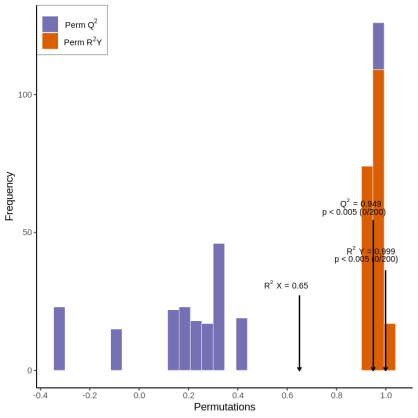


**(C)**


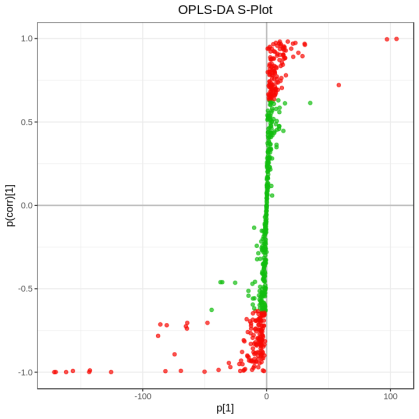

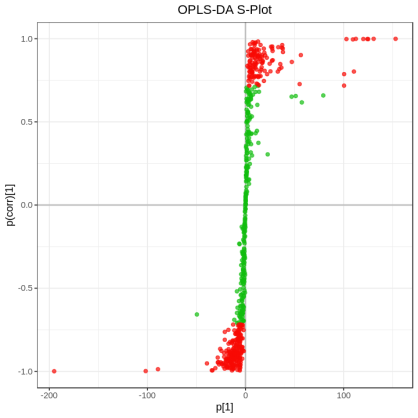

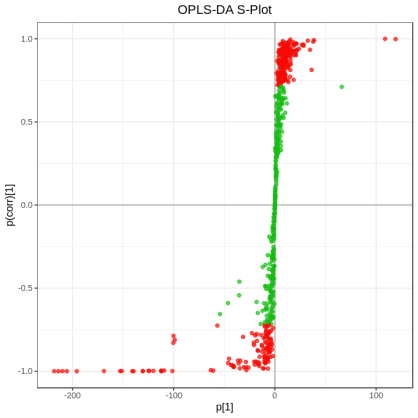


**(D)**


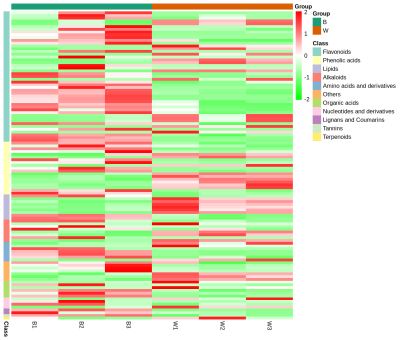

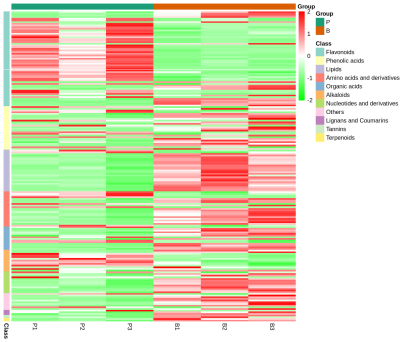

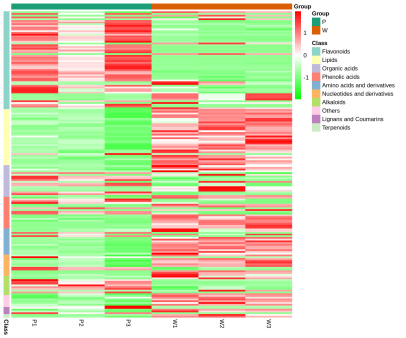

Supplement: Supplementary Figure 1 — (A) Total ion current of one quality control sample by mass spectrometry detection and (B) multi-peak detection plot of metabolites in the multiple reaction monitoring mode. The abscissa represents the retention time (min) of metabolite detection, and the ordinate represents the intensity of the ion current (cps: count per second). (C) The overall cluster diagram of the sample. [file Data_Sheet_1.zip › Supplementary Figure 3.docx]

**Supplementary Figure 4.**

**(A)**


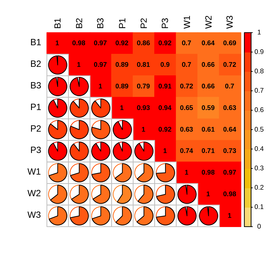


**(B)**


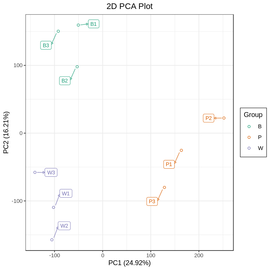


**(C)** (From left to right: BvsW, PvsB, and PvsW)

**
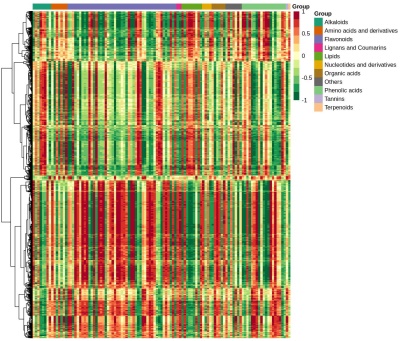

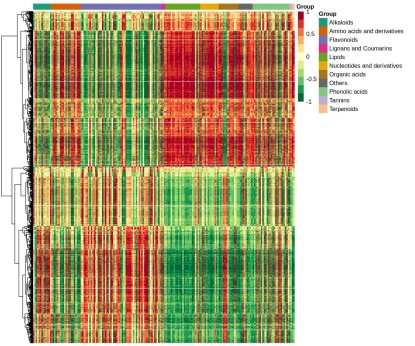

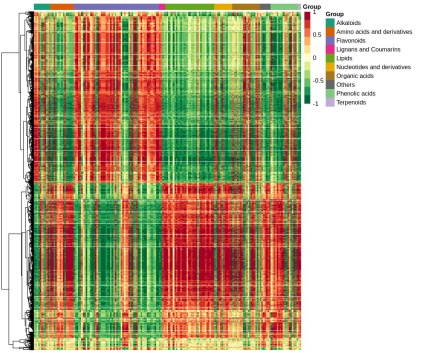
**

Supplement: Supplementary Figure 1 — (A) Total ion current of one quality control sample by mass spectrometry detection and (B) multi-peak detection plot of metabolites in the multiple reaction monitoring mode. The abscissa represents the retention time (min) of metabolite detection, and the ordinate represents the intensity of the ion current (cps: count per second). (C) The overall cluster diagram of the sample. [file Data_Sheet_1.zip › Supplementary Figure 4.docx]
